# Improving outcome in SubaraChnoid HEMorrhage wIth nAdroparin (ISCHEMIA): a prospective randomised controlled trial protocol

**DOI:** 10.1136/bmjopen-2024-096555

**Published:** 2025-08-28

**Authors:** Nadine Denneman, Tom C Doorschodt, Bert A Coert, René van den Berg, Charles B L M Majoie, Marcella C A Müller, Saskia Middeldorp, Michiel Coppens, Marinus A Kempeneers, Maud A Tjerkstra, Homeyra Labib, Harssh Verdan Bandral, Martine Baarse, Ewa E Platek, W Peter Vandertop, Dagmar Verbaan, René Post

**Affiliations:** 1 Neurosurgical Center Amsterdam, Amsterdam UMC Location AMC, Amsterdam, The Netherlands; 2 Neurovascular Disorders, Amsterdam Neuroscience, Amsterdam, The Netherlands; 3 Department of Radiology and Nuclear Medicine, OLVG, Amsterdam, The Netherlands; 4 Department of Radiology and Nuclear Medicine, Amsterdam UMC Location AMC, Amsterdam, The Netherlands; 5 Department of Intensive Care Medicine, Amsterdam UMC Location AMC, Amsterdam, The Netherlands; 6 Department of Internal Medicine, Radboudumc, Nijmegen, The Netherlands; 7 Department of Vascular Medicine, Amsterdam UMC Location AMC, Amsterdam, The Netherlands

**Keywords:** Neurosurgery, Stroke, Anticoagulation, Cerebral Hemorrhage, Mortality

## Abstract

**Introduction:**

Aneurysmal subarachnoid haemorrhage (aSAH) is a severe condition associated with significant morbidity and case fatality rate. Delayed cerebral ischaemia (DCI) is a major factor contributing to poor outcomes. For long, DCI was thought to be caused by vasospasm, induced by blood in the subarachnoid space. Growing experimental and clinical evidence has shown an activation of the coagulation cascade and several other (intravascular) pathophysiological pathways, affecting the cerebral microcirculation. In a retrospective analysis of our aSAH patient registry, we observed lower in-hospital mortality and a significantly higher rate of discharge-to-home in patients treated with high-dose nadroparin, compared with patients treated with low-dose (prophylactic) nadroparin. This observation suggests a potential benefit of higher doses of nadroparin in the acute course after aSAH. We therefore hypothesise that treatment with high-dose nadroparin will improve clinical outcome in endovascularly treated patients with aSAH.

**Methods and analysis:**

This is a single-centre, prospective, phase II randomised controlled trial. From January 2022, all eligible patients will be recruited. 100 patients will be randomised to the intervention arm, that is, nadroparin two times per day 5700 AxaIU, or the control arm, that is, nadroparin once daily 2850 AxaIU in patients with body weight ≤100 kg or once daily 5700 AxaIU in patients with body weight >100 kg, both for up to 21 days. The trial includes a 6-month follow-up period. The primary objective is 30-day mortality rate. Secondary outcomes include assessment of DCI, complications during admission, discharge location, clinical outcome (modified Rankin Scale), quality of life and total healthcare costs at 3 and 6 months follow-up.

**Ethics and dissemination:**

Approval was obtained from the Medical Research Ethics Committee of the Amsterdam UMC, location Academic Medical Center (AMC) (MREC-number 2020_192), and recruitment has begun. The study results will be submitted for publication in peer-reviewed journals and presented at international conferences.

**Trial registration number:**

NCT04507178.

**STRENGTHS AND LIMITATIONS OF THIS STUDY**

- Use of randomised controlled design minimises selection bias and enhances internal validity.
- Stratified randomisation by World Federation of Neurosurgical Societies score ensures balance in clinical severity between groups.
- Single-centre design may limit generalisability to broader patient populations and healthcare settings.
- Blinded outcome assessment reduces risk of detection bias for follow-up measurements.
- Absence of interim analysis may delay detection of potential harm or benefit during trial progression.

## Introduction

 Aneurysmal subarachnoid haemorrhage (aSAH) is a severe type of stroke associated with high rates of morbidity and case fatality rate. Approximately 33% of patients do not survive within 3 months after the haemorrhage, and 20% remain significantly dependent on others for daily care and activities.^1^ Given that half of these patients are younger than 55 years old, the impact on productive life years is substantial, affecting both economic and social aspects.^2^

One of the major complications of aSAH is delayed cerebral ischaemia (DCI), which occurs in 20–40% of patients and can result in brain infarction, permanent brain damage and poor clinical outcomes.^2^,^4^ Cerebral vasospasm, in reaction to aneurysm wall rupture and subarachnoid blood, was long considered to be the principal determinant contributing to DCI. This concept has led to routine treatment with nimodipine, a calcium channel blocker, with only modest success on the prevention of DCI and clinical outcome.^5^ Growing experimental and clinical evidence has shown that not necessarily vasospasm, but the activation of several key pathophysiological pathways may be the principal determinant of DCI. Cortical spreading depressions, endothelial dysfunction, procoagulant activity causing microthrombosis, neuroinflammation, oxidative stress, necrosis and apoptosis may all contribute to brain injury after the acute intracranial circulatory arrest of the initial haemorrhage.^6^,^9^ Due to this partly understood complex pathophysiology, many different treatment strategies have been proposed, of which none seem sufficient for preventing or treating secondary brain damage.^6^

**Figure 1:**
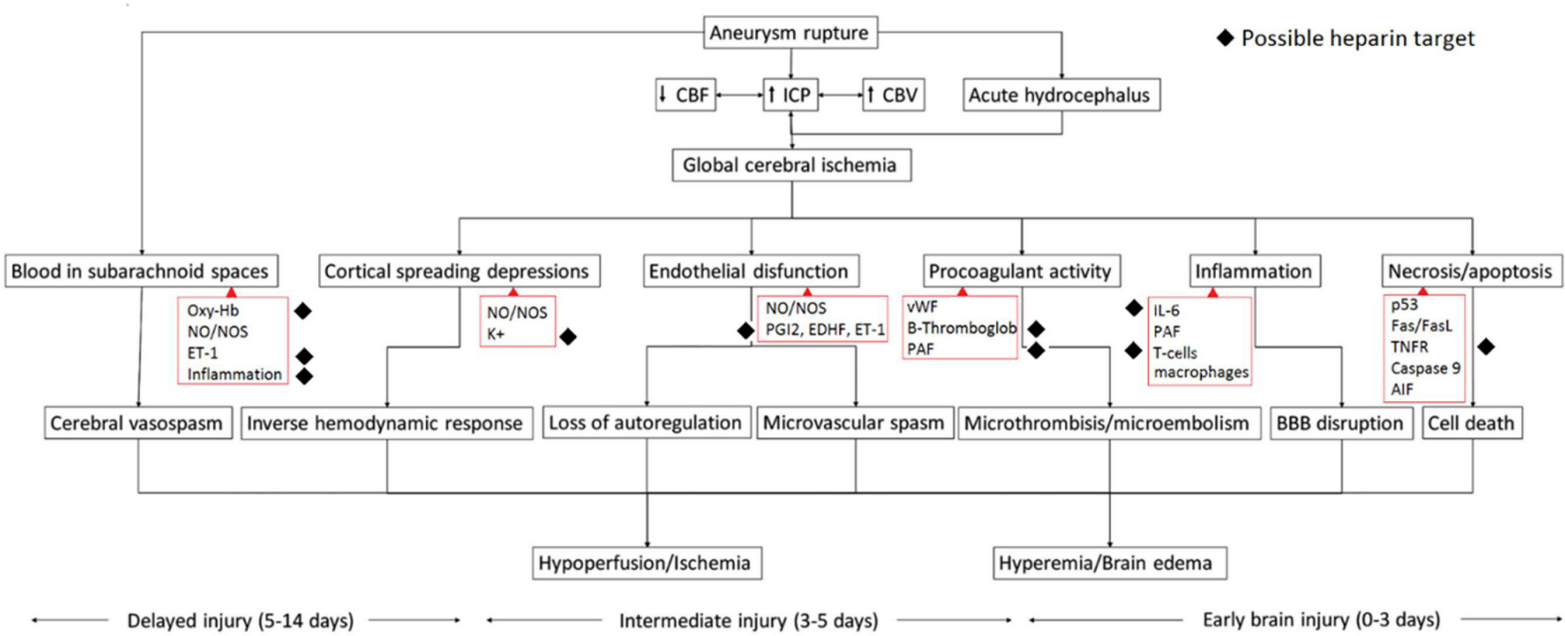
Activation of pathways after aneurysmal rupture and possible heparin targets (adapted image from Budohoski *et al*).^6^ BBB, blood-brain barrier; CBF, cerebral blood flow; CBV, cerebral blood volume; EDHF, endothelium-derived hyperpolarizing factor; ET-1, endothelin-1; ICP, intracranial pressure; IL-6, interleukin-6; K+, potassium; NO, nitric oxide; NOS, nitric oxide synthase; Oxy-Hb, oxyhaemoglobin; PAF, platelet-activating factor; PGI2, prostacyclin; TNFR, tumour necrosis factor receptor; vWF, von Willebrand factor.

Several studies, including one conducted recently in 2021, have argued the possible beneficial effect of heparin in patients with aSAH.^10^,^19^ Recent studies highlight that heparin has extensive anti-inflammatory and immune-regulating properties, which are independent of its anticoagulant effects.^20 21^ Among others, heparin binds oxyhaemoglobin, blocks the activity of free oxygen radicals, antagonises endothelin-mediated vasoconstriction, binds to several cytokines and all chemokines (anti-inflammatory) and several growth factors (antimitogenic and antifibrotic).^20^,^23^ In addition, heparin antagonises the activation of pathways that seem responsible for ischaemic brain damage in patients with aSAH (figure 1). Henceforth, not DCI prevention in itself, but rather modulation of several pathophysiological processes could be targeted by heparin treatment.

One observational study investigated the impact of postinterventional continuous intravenous unfractionated heparin (UFH) in patients with aSAH who underwent endovascular treatment. Findings indicated that the patients receiving therapeutic UFH experienced fewer instances of vasospasm and DCI during their hospital stay compared with those treated with prophylactic low-molecular weight heparin (LMWH).^24^ In contrast, there was no beneficial effect on outcome after 6 months of follow-up. However, as patients with therapeutic UFH were only treated for 7 days, and DCI continues to develop until 14 days after aneurysm rupture, this study could have underestimated the effect of heparin.

We retrospectively analysed patients with aSAH who were treated with high-dose LMWH (nadroparin 5700 AxaIU two times per day until discharge with a median duration of 17 days) and found a significant reduction of in-hospital mortality compared with patients treated with low-dose LMWH (nadroparin 2850 AxaIU once daily until discharge) (5% and 23%, respectively).^25^ In addition, discharge to home was significantly higher in patients who received high-dose LMWH, compared with low-dose LMWH (40% and 17%, respectively). In summary, some studies suggest that higher doses of heparin could improve the clinical outcomes for patients with aSAH, but high-level evidence is lacking. To address this gap, a randomised controlled trial (RCT) is necessary. This phase II trial aims to evaluate the efficacy and safety of high-dose nadroparin combined with standard care, providing essential evidence to determine whether to proceed to a larger phase III trial.

Nadroparin was selected as the investigational product over other LMWHs or UFH based on both practical and theoretical considerations. In our institution, therapeutic use of UFH requires intensive monitoring and titration, which can only be performed in the intensive care unit (ICU) setting. This would necessitate prolonged ICU admission—up to 3 weeks—for all study participants, making it logistically and ethically unfeasible. Furthermore, achieving stable anticoagulant levels with UFH is notoriously difficult in critically ill patients, with the activated partial thromboplastin time often failing to accurately reflect heparin activity, thus increasing the risk of both underdosing and overdosing.^26 27^ Nadroparin, by contrast, has a more predictable pharmacokinetic profile, can be administered subcutaneously and allows for reliable monitoring via anti-Xa levels. Additionally, the potential non-anticoagulant, pleiotropic effects of heparins—such as modulation of neuroinflammation and apoptosis—are expected to be shared across both UFH and LMWHs.^22^ Thus, nadroparin was chosen as the most suitable agent to evaluate the therapeutic potential of heparins in this vulnerable patient population. We hypothesise that treatment with high-dose nadroparin significantly reduces 30-day mortality compared with standard care. To determine this potential benefit, we will conduct an RCT titled ‘Improving outcome in SubaraChnoid HEMorrhage wIth nAdroparin’ (ISCHEMIA). The ISCHEMIA trial challenges existing treatment paradigms, which are mostly aimed at blood-induced vasospasm and will be the first RCT to investigate the effect of high-dose LMWH in patients with aSAH.

## Methods and analysis

### Study design and setting

**Figure 2:**
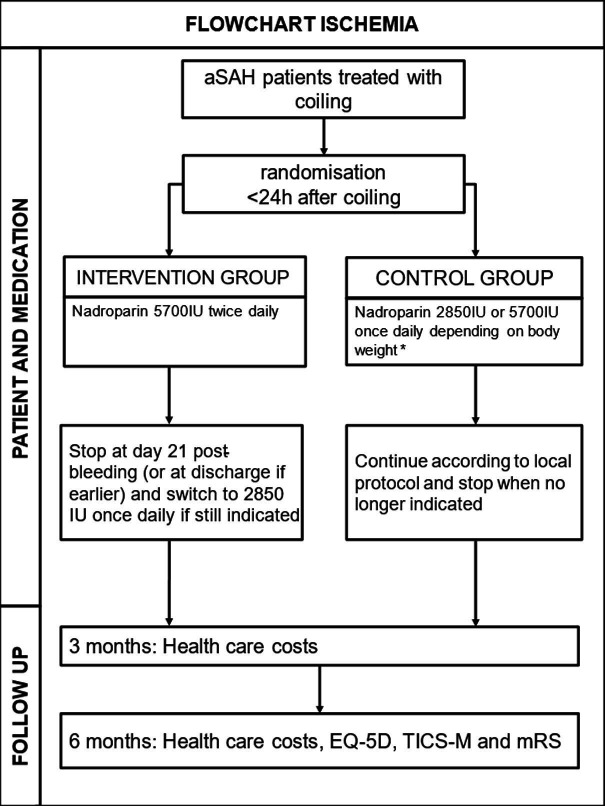
Flowchart. *Once daily 2850 AxaIU in patients with body weight ≤100 kg or once daily 5700 AxaIU in patients with body weight >100 kg. aSAH, aneurysmal subarachnoid haemorrhage; EQ-5D, EuroQol 5-Dimensions questionnaire; ISCHEMIA, Improving outcome in SubaraChnoid HEMorrhage wIth nAdroparin; IU, international units; mRS, modified Rankin Scale; TICS-M, Modified Telephone Interview for Cognitive Status.

This trial is a single-centre phase II prospective randomised trial designed to evaluate the efficacy and safety of high-dose nadroparin combined with standard care in patients with aSAH following endovascular coiling. Eligible patients are randomly assigned to the intervention or control group after written informed consent is obtained. The trial pathway is illustrated in figure 2. The trial protocol has been registered in the National Institutes of Health ClinicalTrials database (number NCT04507178). The trial started randomisation in January 2022 and aims to include 100 patients within 4 years.

### Participants

The study population consists of patients with aSAH admitted to the Amsterdam UMC location University of Amsterdam. Patient selection is based on the following inclusion and exclusion criteria.

#### Inclusion criteria

1. Spontaneous aSAH confirmed by CT or lumbar puncture with the causative aneurysm confirmed by CT-angiography and/or digital subtraction angiography.
2. Coiling of the causative aneurysm within 72 hours of initial SAH.
3. Informed consent before the first scheduled administration of the study medicine.
4. Adult age (≥18 years).

#### Exclusion criteria

1. Stent-assisted coiling.
2. Use of anticoagulant or dual antiplatelet medication postcoiling for other indications.
3. Incomplete aneurysm occlusion/partial coiling.
4. Intraparenchymal haemorrhage.
5. Arteriovenous malformation.
6. Pregnancy.
7. Contraindications for LMWH:
  - History of heparin-induced thrombocytopenia.
  - (Suspicion of) active arterial or venous bleeding.
  - History of haemorrhagic diathesis due to coagulation disorders (with the exception of disseminated intravascular coagulation)
  - Severe hypertension: uncontrolled hypertension with a persistent mean arterial pressure (MAP) >135 mm Hg.
  - History of hypertensive or diabetic retinopathy.
  - History of active infectious endocarditis.
  - Severe renal impairment (creatinine clearance <30 mL/min).
8. Patient or substitute decision-maker (SDM) lacks proficiency in Dutch and English.
9. Proven and active COVID-19 infection.

### Recruitment and consent

If patients are eligible for the study, they, or their legally appropriate SDM, will be informed about the study’s rationale, possible risks and study burden. A sample consent form is provided in online supplemental appendix C. To optimally modify several key pathophysiological intravascular factors, it is paramount that the study medication is started as early as possible, at least within 24–72 hours after coiling. Therefore, there will be a maximum reflection period until the first scheduled administration of the study medication (mostly within 24 hours after the coiling procedure, unless other reasons necessitate postponement; see section ‘Interventions’). Subjects, or their legally appropriate SDM, who have given consent for inclusion in the study can decide to exit the study at any time for any reason without any consequences.

### Allocation and randomisation

After obtaining informed consent, patients will undergo randomisation with stratification for World Federation of Neurosurgical Societies (WFNS) score. Five strata were defined, one for each WFNS score. Within each stratum, separate randomisations are performed between the intervention and control group to ensure a balanced randomisation based on disease severity. This is particularly important because, in patients with high-grade WFNS scores, goals of care may shift toward comfort-focused measures, which can independently affect mortality outcomes regardless of the intervention. Stratification by WFNS grade helps mitigate this potential source of bias. Randomisation is conducted using a web-based permuted block system and performed by the principal investigator or executive researcher. This system employs permuted blocks of variable sizes, ranging from two to six patients, to minimise the predictability of group allocation and ensure balanced sample sizes in each study arm. Patients are randomised in a 1:1 ratio and assigned to one of the two treatment arms.

### Interventions

Patients in the intervention group will receive a high dose of LMWH, specifically nadroparin 5700 AxaIU administered two times per day. Treatment will start as soon as the redeemed safe is available, but no later than 24–72 hours after coiling and will continue for 21 days following the initial SAH. For safety reasons, high-dose nadroparin will only be administered to patients with a secured aneurysm. The current inclusion criteria consider both the time of ictus and the timing of coiling. Only patients who undergo coiling within 72 hours of ictus are eligible for inclusion, with most starting nadroparin within 24 hours after coiling. In clinical practice, the majority of eligible patients present on the day of ictus or the following day and are coiled within 24 hours. Since DCI typically develops between days 4 and 10 after SAH, this time frame is considered an appropriate therapeutic window. If discharged home before 21 days, the treatment will stop on discharge. If hospitalisation extends beyond 21 days, the prophylactic dose will be continued until discharge or until the patient is mobilised for more than 6 hours per day. The high dose of nadroparin used in this study is solely considered therapeutic in patients weighing under 70 kg. This fixed dosing approach reflects the study’s focus not solely on anticoagulant effects but also on the broader pleiotropic properties of LMWH, such as anti-inflammatory and endothelial-stabilising actions. In general, target anti-Xa levels for nadroparin are unknown. Based on observed anti-Xa levels in subjects with a therapeutic weight-based dose, reference ranges are regularly around 0.6–1.2 IU/mL. However, it remains uncertain whether benefits and harms of nadroparin are optimally maintained in this range.^28^

In the control group, patients will receive standard care with a prophylactic dose of LMWH (nadroparin; 2850 AxaIU once daily for those weighing ≤100 kg, or 5700 AxaIU once daily for those weighing >100 kg), starting within 24 hours after coiling and continuing until discharge or when mobilised for at least 6 hours daily.

Patients with anticoagulants receive reversal agents on admission as standard care. In the case of vitamin K antagonists and heparin derivatives, patients in the intervention group will only start with high-dose LMWH after receiving reversal agents, with an international normalised ratio ≤1.5, and 24 hours after the initial bleeding (unless a craniotomy is indicated). In the case of direct oral anticoagulants (DOACs) use, high-dose LMWH will not be administered until 48 hours after receiving the last dose of DOAC since there are often limited options to reverse its action. Antiplatelets are often not counteracted unless a craniotomy is performed. In the case of antiplatelet use, high-dose LMWH will be postponed until 24 hours after the initial bleeding. In the case of dual antiplatelet therapy, a cardiologist will be consulted to assess whether it’s possible to switch to high-dose LMWH. If this is not possible, the patient will be excluded. The investigator or treating physician can decide to withdraw a subject from the study for urgent medical reasons. If it is revealed after inclusion that one of the exclusion criteria was present in a certain patient on admission, this patient will remain included in the study and this is recorded as a protocol violation. Depending on the criterion, actions are undertaken. For instance, nadroparin can be stopped (history of heparin-induced thrombocytopenia, history of hypertensive or diabetic retinopathy, endocarditis) or switched to an alternative type of LMWH or the higher dose will be switched for a prophylactic dose (severe renal impairment, intestinal tract bleeding). If nadroparin is not available or not tolerated, mostly due to skin reactions, other LMWHs can be administered depending on local availability and according to the doses listed in online supplemental appendix A. In case of mild renal failure (creatinine ≥30 mL/min and <50 mL/min), the dose of LMWH in the intervention arm will be reduced by 25%. Emergency surgical procedures (eg, lumbar puncture, insertion of ventricular catheter or lumbar drain) and procedures for bleeding in patients receiving a higher dose of LMWH conform to standard routine clinical care. A flowchart is included in online supplemental appendix B.

### Outcomes

The primary objective is to evaluate whether treatment with high-dose LMWH in patients with coiled aSAH significantly reduces mortality assessed at 30 days after the initial bleeding. Secondary objectives are to evaluate whether this treatment significantly reduces the presence and severity of DCI (measured by the Modified National Institutes of Health Stroke Scale),^29^ increases the incidence of major bleeding (defined as a fatal bleeding and/or symptomatic bleeding in critical areas or organs (such as intracranial, intraspinal, intraocular, retroperitoneal, intra-articular, pericardial or intramuscular bleeding causing compartment syndrome) and/or bleeding resulting in a haemoglobin drop of 1.2 mmol/L or more, or requiring a transfusion of two or more units of whole blood or red cells) and non-major bleeding (defined as all reported bleedings not classified as major),^30^ increases the risk of haemorrhagic complications after shunt placement or lumbar puncture, reduces other SAH-related complications (such as severe hyponatraemia, postprocedural aneurysm rupture, rebleeding, delirium, epilepsy, diffuse parenchymal swelling or nosocomial infections), reduces the incidence of shunt-dependent hydrocephalus, increases the rate of discharge to home, improves quality of life at 6 months, enhances cognitive functioning at 6 months, increases the likelihood of a favourable outcome at 6 months and reduces mortality at 6 months. Outcome assessments are done by a trial nurse at the outpatient clinic 6 months after the initial bleeding, with the standard follow-up performed on the same day. The trial nurse is blinded for group allocation and does not participate in the medical treatment of the included patients. In addition, peak anti-Xa levels are measured at 12:00 on day 7 following the ictus and are readily available for analysis. A comprehensive panel of coagulation and inflammatory biomarkers is also being collected and will be analysed after completion of patient inclusion. The biomarkers include von Willebrand factor, syndecan-1, soluble thrombomodulin, tumour necrosis factor-alpha, interleukin (IL)-6, IL-8, soluble vascular cell adhesion molecule-1, angiopoietin, a disintegrin and metalloproteinase with thrombospondin motifs 13, D-dimer and coagulation factor III (also known as tissue factor). All biomarker measurements are performed on day 7 following the ictus.

### Sample size calculation

In our retrospective analysis, we found an 18% reduction in mortality when patients were treated with high-dose LMWH (23% mortality in the low-dose LMWH group versus 5% mortality in the high-dose LMWH group, OR 0.19, 95% CI: 0.07 to 0.55).^25^ Although this is a substantial reduction, it was derived from a retrospective, non-randomised design, which limits the ability to assess causal inference and make proper adjustments for confounding prognostic variables. Since we cannot assume this effect size to be accurate without further validation, we aim to conduct a phase II RCT with a total of 100 patients (50 patients per group). After the inclusion and follow-up of these patients, we hope to perform a more reliable power calculation to proceed to a phase III trial.^31^ Besides clinical data, this study will help evaluate the financial and logistical feasibility (including compliance to the study medication, data collection and costs) of establishing a full-scale study.

Each year, 120–125 patients with aSAH are admitted to the Amsterdam UMC, with 80–90 of these patients receiving coiling of the causative aneurysm. Considering the exclusion criteria, we estimate that roughly 25–50 patients per year could be eligible for the ISCHEMIA trial.

### Statistical analysis

All enrolled patients will be evaluated for the primary and secondary outcomes using an intention-to-treat analysis. To ensure the robustness of the findings, a per-protocol and as-treated analysis will also be conducted, excluding participants who do not complete follow-up, switched treatment groups, had major protocol violations and/or non-adherent patients. Baseline characteristics will be summarised by means of simple descriptive statistics. Continuous data with a parametric distribution will be presented as mean and SD, while non-parametric data will be presented as median and IQR. For between-group comparisons, independent samples t-tests or Mann-Whitney U tests will be used as appropriate. Categorical data will be presented as percentages, with the χ² test used for between-group comparisons.

The treatment effect on the primary outcome (30-day mortality) will be analysed using multivariable logistic regression, adjusting for any clinically relevant baseline imbalances if necessary. Secondary outcomes will be analysed using independent samples t-tests, Mann-Whitney U tests, χ² tests and multivariable linear and logistic regression models, as appropriate. Sensitivity analyses will also be performed, allowing early withdrawal of life support to be considered as a potential confounding variable.

All statistical uncertainty will be expressed using 95% CIs. Statistical analyses will be performed using SPSS (IBM, Armonk, New York, USA), with a significance level set at p<0.05, and all tests will be two-sided.

### Data safety analysis

Adverse events (AEs) and serious AEs (SAEs) will be recorded in our electronic case report form (CRF). Only AEs occurring during hospital admission that are unrelated to the SAH must be reported to the institutional review board and principal investigator. All SAEs will be reported without undue delay after obtaining knowledge of the events. In this trial, certain SAEs are anticipated in patients with SAH, including severe hyponatraemia (serum sodium <125 mmol/L), hypokalaemia (serum potassium <3.5 mmol/L), fever, hydrocephalus, DCI, periprocedural aneurysm rupture, rebleed, delirium, Terson’s syndrome, epilepsy, diffuse parenchymal swelling, nosocomial meningitis, pneumonia and urinary tract infection. These expected SAEs will be recorded in the CRF and do not require further reporting.

A data and safety monitoring board (DSMB) is installed for this trial including a neurologist, neurosurgeon and independent statistician. The members are not involved in the trial and have no competing interests. Their tasks, responsibilities and working procedures are outlined in a charter. The DSMB will review safety data and the incidence of (S)AEs at three specified intervals: After 30, 60 and 100 patients have completed their 30-day follow-up. The DSMB will conduct ongoing safety surveillance, particularly regarding the occurrence of SAEs related to an increased risk of haemorrhagic complications. Additionally, the DSMB will advise on the continuation of the trial and may recommend the Steering Committee of the ISCHEMIA trial to terminate the trial if there is clear and substantial evidence of harm. No interim analysis is planned in this trial.

## Ethics and dissemination

The protocol for this trial received approval from the Medical Research Ethics Committee (MREC) of Amsterdam UMC, location AMC in Amsterdam, the Netherlands (MREC-number 2020_192). The study will adhere to the principles outlined in the Helsinki Declaration. Results from this research will be submitted for publication in a peer-reviewed journal, and presented at national and international conferences.

### Patients and public involvement

Patients were not involved in the design, conduct, reporting or dissemination plans of this research.

## Discussion

The results of this phase II RCT will offer valuable insights into the efficacy and safety of high-dose nadroparin in patients with aSAH treated endovascularly. Our retrospective analysis suggests that high-dose LMWH may improve clinical outcomes by reducing in-hospital mortality and increasing discharge rates to home compared with standard prophylactic doses.^25^ The ISCHEMIA trial is particularly significant as it is the first to rigorously assess the impact of high-dose nadroparin in this patient population through a prospective, randomised design. Priorities of this trial are to further evaluate the clinical efficacy and safety profile of high-dose nadroparin, refine our power calculations and assess the financial and logistical feasibility of implementing this treatment regimen in the future. The additional data and revised methodologies will inform the design and implementation of a full-scale phase III trial. Given the case fatality rate associated with aSAH, we would consider an absolute mortality reduction of 5% or a relative risk reduction of at least 20% to be a clinically meaningful threshold to justify progression to a larger trial.

The anticipated benefits of high-dose nadroparin are based on its broad anti-inflammatory and immunomodulatory effects, extending beyond anticoagulation.^20 21^ By targeting key pathophysiological processes involved in DCI, such as cortical spreading depressions, endothelial dysfunction and neuroinflammation, nadroparin might mitigate secondary brain injury more effectively than current standard treatments. Given the complex and not fully understood mechanisms underlying DCI, this trial addresses an important gap in our therapeutic approach to aSAH. If proven effective, this treatment is expected to lead to improved clinical outcomes, reduced mortality and fewer SAH-related complications.

There are several strengths to this trial. Patients will receive high-dose nadroparin only during their hospital admission and for a maximum of 21 days, ensuring no postdischarge burden. Additionally, nadroparin is relatively inexpensive, suggesting that future implementation would face minimal financial constraints. The study includes both primary and secondary objectives, with secondary outcomes covering a wide range of clinical parameters, offering a comprehensive assessment of the potential benefits and risks of high-dose nadroparin. Rigorous methodology, including stratified randomisation and intention-to-treat analysis, enhances the reliability and validity of the results. By selecting 30-day mortality as the primary outcome, the study employs a clear, objective and clinically significant endpoint, reducing subjective interpretation and providing a definitive assessment of the treatment’s impact.

This trial has some limitations. As a phase II RCT with a small sample size, it lacks the power to provide definitive conclusions about the effect of high-dose nadroparin on mortality and other parameters, necessitating a larger phase III RCT for confirmation. The trial is not fully blinded. We opted for an open-label approach due to both practical and ethical considerations. Nadroparin carries a risk of bleeding, and early recognition of haemorrhagic complications is essential. Unblinding in response to clinical deterioration may delay critical decisions or lead to uncertainty in management. Moreover, the dosing regimens differ between groups: patients in the intervention group receive nadroparin two times per day, while those in the control group receive it once daily. This distinction makes full blinding unfeasible, as it would likely be apparent to both patients and care providers. Additionally, nadroparin has measurable pharmacodynamic effects—such as anti-Xa levels—which would further compromise blinding, as clinicians could infer treatment allocation from laboratory results. Using a placebo control with sham anti-Xa monitoring was considered but deemed both ethically and logistically challenging. Simulating treatment would require drawing blood and performing lab tests solely to maintain blinding, offering no clinical benefit and exposing critically ill patients to unnecessary procedures. To minimise bias, a blinded trial nurse was included, and the primary outcome—mortality—was selected for its objectivity and resistance to observer bias. A further limitation of the study is that not all patients may complete all dosages due to emergency interventions requiring temporary cessation of the study medication, such as lumbar punctures or ventricular catheter placements. The heterogeneity of aSAH patients and the exclusion of those with intraparenchymal haemorrhage or non-endovascular treatments complicate the generalisation of results to the broader aSAH population or to settings preferring different aneurysm occlusion methods, such as clipping. Preferably, a larger and more diverse population will be included in future trials to establish broader applicability, if deemed safe.

In conclusion, the pathophysiology of aSAH and particularly DCI is complex and not well understood. The outcomes of this trial will guide the feasibility and design of a larger phase III RCT, which is essential for establishing the definitive clinical utility of high-dose nadroparin in improving patients with aSAH outcomes. If the hypothesised benefits are confirmed, this study could lead to a paradigm shift in the management of aSAH, moving beyond the current focus on vasospasm prevention to a broader approach targeting multiple pathophysiological pathways. The findings may also have significant implications for healthcare costs and resource allocation, potentially improving both clinical and economic outcomes in this patient population.

## Supplementary material

10.1136/bmjopen-2024-096555 online supplemental file 1

10.1136/bmjopen-2024-096555 online supplemental file 2

10.1136/bmjopen-2024-096555 online supplemental file 3
