# Supplementary material for: Improving outcome in SubaraChnoid HEMorrhage wIth nAdroparin (ISCHEMIA): a prospective randomised controlled trial protocol
Source: BMJ Open. 2025 Aug 28;15(8):e096555. doi: 10.1136/bmjopen-2024-096555 (PMC12410639; doi:10.1136/bmjopen-2024-096555)
Supplement: online supplemental file 1 [file bmjopen-15-8-s001.pdf]

Information letter regarding medical research participation

ISCHEMIA

Improving outcome in SubaraChnoid HEMorrhage wIth NAdroparin

Introduction

Dear Sir/Madam,

With this letter you are asked to participate in in medical research. Participation is always on a voluntary basis. The reason you receive this letter is that you've had a hemorrhagic stroke due to a weak spot at an artery in your brain that has ruptured.
This letter provides information about the study mentioned above, what this means for you, and the possible benefits and disadvantages of participation. We understand that this is a lot of information. We would like to ask you to read the information and to decide whether you wish to participate. If you do decide to participate, please fill in the form in Appendix E.

Ask questions

You can base your decision upon the information provided in this letter. In addition we advise you to:
- Ask remaining questions to the researcher(s) providing you the information.
- Discuss the information in this letter with your partner, friends or family.
- Ask questions to dr. Mariam Slot, an independent doctor who is not involved in conducting the study.
- Read the information on https://www.government.nl/topics/medical-research

1. General information

This study is coordinated by the Amsterdam UMC, location AMC, from here on named the initiator of this trial. This trial is conducted by trained and qualified physicians, researchers and research nurses. The medical ethics committee of the AMC has approved this trial.

2. What is the goal of the trial?

We are comparing the function and safety of high-dose nadroparin (5700 IE twice daily) to low-dose nadroparin (2850 IE or 5700 IE depending on body weight once daily). Nadroparin is internationally administered since many years for prevention (low dose: 2850 IE) and treatment (high dose: 5700 IE) of thrombosis.

3. Background of the trial

You have had a hemorrhagic stroke due to a weak spot at an artery in your brain that has ruptured. This bleeding is called a subarachnoid hemorrhage, abbreviated to SAH. The weak

spot is called an aneurysm. Usually, the treatment for an aneurysm is a coiling procedure: a small catheter is inserted through the femoral artery to the aneurysm after which the aneurysm is occluded with tiny wires (=coils). This prevents the aneurysm from bleeding again.
After the hemorrhage and coiling of the aneurysm there's a period in which complications may occur. The most important, and least understood, complication is the occurrence of cerebral infarction. Through a complex, and partially unknown process, there can be reduced blood flow to certain parts of the brain. Decreased blood flow and oxygen to the brain can lead to an infarction, which causes irreversible brain damage. It is important to prevent these infarctions from occurring in order to prevent irreversible damage.
Nadroparin is an anticoagulant, also known as a “blood thinner”, which is being used for many decades by hospitals worldwide as standard practice to prevent and treat blood clots. Previous studies indicate that a higher dose of nadroparin could help reduce the damage caused by an infarction. With this trial we want to investigate whether a high dose of nadroparin can lead to less brain damage and thus better outcome in patients suffering from SAH.

4. How is the trial organized?

How long does the trial take?
The trial takes a total amount of six months to complete, from beginning to end.

Step 1: Are you eligible for the study?
First, we would like to know if you're eligible for the study. To decide this the investigator will look at your medical history.

Step 2: The treatment
In this trial we divide patients into two groups:

- Group 1. Patients in this group receive the high dose nadroparin, for a total amount of 21 days. After this, the nadroparin is switched to low (regular) dose if still medically indicated. Upon discharge from the hospital the nadroparin is stopped, even if this is within the first 21 days.
- Group 2. Patients in this group receive low (regular) dose nadroparin, for as long as medically indicated. Upon discharge from the hospital the nadroparin is stopped.

Your draw will decide which treatment you'll receive.

Step 3: Investigations and measurements
For a clear flow diagram see Appendix C.
The largest part of the trial takes place during hospital admission, which is three weeks on average.
Besides the regular medical care we perform the following investigations:

- Blood sampling. One of the nurses will draw blood between day 6-8 after admission. Blood samples are only taken once, and the maximum amount of blood drawn is 15 ml. This amount of blood is very small and is associated with negligible risk. For comparison: people donating blood to the blood bank donate 500 ml each time. The blood samples are used to test the efficacy of the given medication (nadroparin) and the effect on blood clotting and markers of inflammation.
- At about 3 and 6 months after your bleeding, you receive a short questionnaire with questions that deal with medical expenses caused by the bleeding and quality of life at that time. It takes about 10 minutes to finish the questionnaire. You can choose between a paper or a digital version. If you choose the paper version, you can return the questionnaire by a return envelope.
- Six months after the bleeding a telephone appointment is scheduled. During this appointment a questionnaire and cognition test will be taken. Furthermore, in this period an MRI of the brain will be scheduled. This is standard care for every SAH patient.

Step 4: Follow-up
Six months after the bleeding you will be called by a nurse who does not know whether you received the high dose or low dose nadroparin. This nurse should not be told which dose you received to ensure a reliable assessment about your clinical situation. The nurse will take a brief interview about your functioning at that time and the care and/or medication you have been needing after hospital admission.

What is different compared to regular care?
There is not much different in this trial compared to standard care.

5. What are the agreements?

We would like the trial to go as smooth as possible. Therefore, we ask you to adhere to the following:

- During participation in this trial you don’t participate in another interventional research trial that could possibly interfere with our study (i.e. another medication study). If you have questions about participating in multiple trials please contact the researchers.
- You come to every appointment.
- You contact the investigator in the following situations:
  - If you wish to withdraw from the study.
  - If your telephone number, address or email address changes.

Is it allowed to become pregnant during the trial?
Pregnant or lactating women cannot participate in this trial. After discharge from the hospital it is allowed to become pregnant again without having to leave the trial.

What if I’m pregnant?

Are you pregnant during the trial? Please contact the investigator as soon as possible. It is important to discuss whether it is needed to quit the trial.

6. What are the possible side effects or disadvantages you might experience?

A higher dose of nadroparin may have side effects or disadvantages.
The following side effects are rare, but could be serious:
- Allergic reaction to nadroparin. This is extremely rare.
- Decrease in the amount of platelets in the blood. This is also very rare.
- Increased risk of bleeding. This can occur at the site of injection but also in other places within the body. Since the trial doesn’t start until after the coiling procedure the risk of another hemorrhage from the aneurysm is virtually nill. The risk of hemorrhages in other parts of the body is also very small, as was proven by our previous study. However, there are strict rules regarding the administration of nadroparin around certain medical procedures and/or operations. In case a hemorrhage does occur, an antidote is used to reverse the effects of nadroparin.

A lower dose of nadroparin can also lead to side effects. The most important being an allergic reaction or a decrease in the amount of platelets in the blood.

More information regarding the side effects of nadroparin is in Appendix D.

7. What are the possible benefits and disadvantages of participation in this study?

Participating in medical research can have benefits and disadvantages. We will summarize them below. It is important to take this into consideration, and talk to others if necessary.

Benefits:
It is unknown whether you will benefit from participation in this study. The researchers do not know whether the higher dosage of nadroparin indeed leads to fewer complications and less brain damage. Previous studies do suggest that a higher dosage leads to less mortality, less irreversible damage and thus a better outcome. This trial will yield valuable information and based on the results it will be decided whether a high dose of nadroparin will be standard care in patients suffering from a subarachnoid hemorrhage in the future.

Participation can have the following disadvantages:
- You might suffer from side effects or adverse effects of nadroparin, as described in paragraph 6.
- You might suffer from the measurements that are conducted during the trial. For example: a blood draw might lead to some, albeit mild, pain. Also, a bruise might occur after blood draw.
- Participation costs you some extra time (i.e. due to the two extra questionnaires).
- You are requested to follow the agreements that are part of the trial.

What happens if you do not want to participate in the study?
Participation in this study is completely voluntary. If you do not want to participate you will receive the standard treatment as for all patients with a subarachnoid hemorrhage. This involves the regular dose of nadroparin: 2850 units (body weight < 100 kg) or 5700 units (body weight > 100 kg) once a day. Your doctor can give you further advise regarding the benefits and disadvantages.

8. When does the trial end?

The researcher will inform you in case new information arises that might be of importance to you. Afterwards, the researcher will ask if you want to proceed with participating.

In the following situations your participation will be ended:

- All measurements have been completed.
- You decide to withdraw from the trial. You can always withdraw without reasons. Please inform the researchers of this trial if you wish to withdraw. After withdrawal you will receive the standard treatment for patients with a subarachnoid hemorrhage.
- The researcher decides it is wiser for you to quit the trial.
- One of the following organizations decides to end the trial:
    - The Amsterdam UMC, location AMC
    - The government
    - The medical ethical committee responsible for the ethical review of this trial

What happens if you withdraw from the trial?
The researchers will use the data and blood that has been collected until the moment of withdrawal. If you wish it's possible to destroy the collected blood samples. Please inform the researchers about this.

The trial ends after all measurements of the included patients are finished.

9. What happens after the trial?

After completion of the study the results will be published within approximately two years. You will be informed about the results.

10. What happens to your data?

By participating in this trial you give permission to collect, use and store your medical data and some blood tubes.

What data do we store?
We store the following data:
- your name
- your gender

- your address
- your date of birth
- data concerning your health
- (medical) data collected during the trial

What kind of bodily fluids do we store?
We store blood tubes for a period of five years.

Why do we collect, use and store your data and blood?
We collect, use and store your data and blood to answer the questions proposed in this research, and to be able to publish these results.

How do we protect your privacy?
To protect your privacy we replace your personal information by a code. This code is placed on all your data and blood tubes. The key to this code is stored in a safe place within the hospital. Only the study's researchers have access to the code. When working with this data, solely the code is used. Also when the study results are published, your personal information is kept confidential.

Who can access your data?
A few people have access to your name and other personal data. These are people that inspect the integrity of the research. The following people can access your personal data:

- Representatives of the Health Care Inspectorate can be given access to the medical file to inspect the integrity of the research.
- A monitor assigned by the project leader of the research. This inspection will take place under the responsibility of the treating physician.
- National authorities such as the Health and Youth Care Inspectorate.

The people that have access to your data will maintain confidentiality. We kindly ask your permission for their inspection.

How long do we store your data and blood?
The study data are kept for 15 years within the hospital. Blood tubes are stored within the hospital for five years. Afterwards, the data will be destroyed.

Can we use your data and blood for other studies?
Your data and (remaining) blood might be of value for other studies focusing on brain hemorrhage or the use of nadroparin, after this study has finished. For this reason, your data will be stored for 15 years and blood will be stored for five years. On the consent form you can indicate whether or not you give permission to use your data for other studies. If you don't wish to give permission you can still participate in this study. You will receive the same care.

What happens when an unexpected finding occurs?

During this study we might find something unexpected that could be of importance to your health. If this occurs the researcher will inform your general practitioner. You and your general practitioner or treating physician will discuss what to do next. By participating in this study you give permission to inform your general practitioner or treating physician during any unexpected medical events.

Can you withdraw your permission for using your data?
You can withdraw your permission at any time. However, if you decide to withdraw after your data has been collected the researchers are still allowed to use the collected data. The blood samples will be destroyed after you decide to withdraw, unless your blood has already been analyzed. If this is the case the researchers are still allowed to use these measurements.

Do you want to know more about your privacy?

- If you wish to know more about your rights concerning the use of personal information, you can visit https://www.autoriteitpersoonsgegevens.nl/en.
- Do you have questions regarding your rights? Or do you have a complaint regarding the processing of your personal data? Please contact the person responsible for the use of personal data. This is:
    - Amsterdam UMC, location AMC. See Appendix A for the contact details, and website.
- If you have a complaint regarding the processing of your personal data we advise you to discuss this with the research team first. You can also visit the data privacy officer of the Amsterdam UMC, location AMC. Another option is to file a complaint at the Dutch Data Protection Authority.

Where can you find more information regarding this study?
On the following website you can find information regarding this study: www.clinicaltrialsregister.eu. After the study has finished the website will provide a summary with the results of the study. You can find the study by searching for ‘ISCHEMIA’ (number: 2018-000790-79)

11. Do you receive compensation for participating in this study?

The extra measurements and treatment during the study do not cost you any money. You will not receive compensation for participating in this study.

12. Are you insured during the study?

For all participants in medical research an insurance is taken out. The insurance is taken out in order to cover for damage to the research subjects. However, not all types of damage are covered. More about the insurance and the exceptions for damage coverage can be found in appendix B. Here you can also find whom to contact.

13. We inform your general practitioner and/or treating physician and/or pharmacist

The researcher will inform your general practitioner about your participation. This is for your own safety. If there are questions concerning your medical history and/or medication use we might contact your general practitioner or treating physician.

14. Do you have questions?

You may ask questions related to the study to your treating physician. If you wish to receive advise from someone not involved in this study you may speak to dr. K.M. Slot, neurosurgeon in the Amsterdam UMC, location AMC. She an independent doctor who is not involved in conducting the study, but knows a lot about performing research.
Do you have a complaint? Please discuss this with the researcher or your treating physician. If you don't wish to speak to one of the people mentioned above you may also go to the complaints committee or the Official for Data Protection of the Amsterdam UMC, location AMC. See Appendix A for the contact details.

15. How do you give informed consent?

When you have had enough time to consider participation in this study you can tell the researcher or physician that you understand the information in this letter and that you give or refuse permission for participation in the study. Do you wish to participate? Please fill in the consent form that is attached to this information letter. You and the researcher will both receive a signed version of this consent letter.

We thank you for reading this information letter.

16. Appendices

A. Contact details

B. Information regarding insurance

C. Flowchart of the study

D Side effects, adverse effects and disadvantages of nadroparin

E Consent form participant

Appendix A: Contact details for the Amsterdam UMC, location AMC

Principal investigators

Dr. D. Verbaan, associate professor and clinical epidemiologist (Amsterdam UMC, location AMC)

The contact details have been redacted for privacy.

Prof.dr. W.P. Vandertop, chairman and neurosurgeon (Amsterdam UMC, location AMC)

The contact details have been redacted for privacy.

Independent physician:

Dr. K.M. Slot, neurosurgeon (Amsterdam UMC, location AMC)

The contact details have been redacted for privacy.

Complaints:

"Afdeling Patiëntvoorlichting & Klachtenopvang" Amsterdam UMC, location AMC

The contact details have been redacted for privacy.

Official for Data Protection of the AMC:

Mr. J.B. Inge

The contact details have been redacted for privacy.

For more information regarding your rights:

Amsterdam UMC, location AMC, tel no: 020-566911, www.amc.nl.

Appendix B: Information regarding insurance

For all participants in this study an insurance is taken out by the Amsterdam UMC, location AMC. The insurance covers damage caused by participation in this study. This applies to damage that occurs during the study until 4 years after the end of your participance. Any damage must be reported to the insurer within 4 years.

Not all damage is covered by the insurance. At the bottom of this paragraph you can find what is not covered by your insurance.
These terms are stated in the 'Besluit verplichte verzekering bij medisch-wetenschappelijk onderzoek met mensen 2015' and can also be found in the law database (‘Wettenbank’) of the government (https://wetten.overheid.nl).

In case of damage you can directly contact the insurer.

The insurer for this study is:
Name: Centramed
Address: PO Box 7374, 2701AJ, Zoetermeer
Telephone number: 070 301 70 70
E-mail: schade@centramed.nl
Policy number: 620.872.806

The insurance covers €650.000 per participant, €5.000.000 for the complete trial and €7.500.000 for all trials from the same client.

The insurance does **not** cover the following damage:
- Damage caused by a risk you have been informed about in the written information. This does not apply if the risk is more severe than was anticipated or if the occurrence was deemed highly unlikely;
- Damage to your health that would have also occurred without participance in this study;
- Damage caused by not following the instructions or recommendations;
- Damage to your offspring, caused by a negative effect of the study to you or your offspring;
- Damage caused by an existing treatment method in research concerning existing treatment methods.

Appendix C: Flowchart of the study

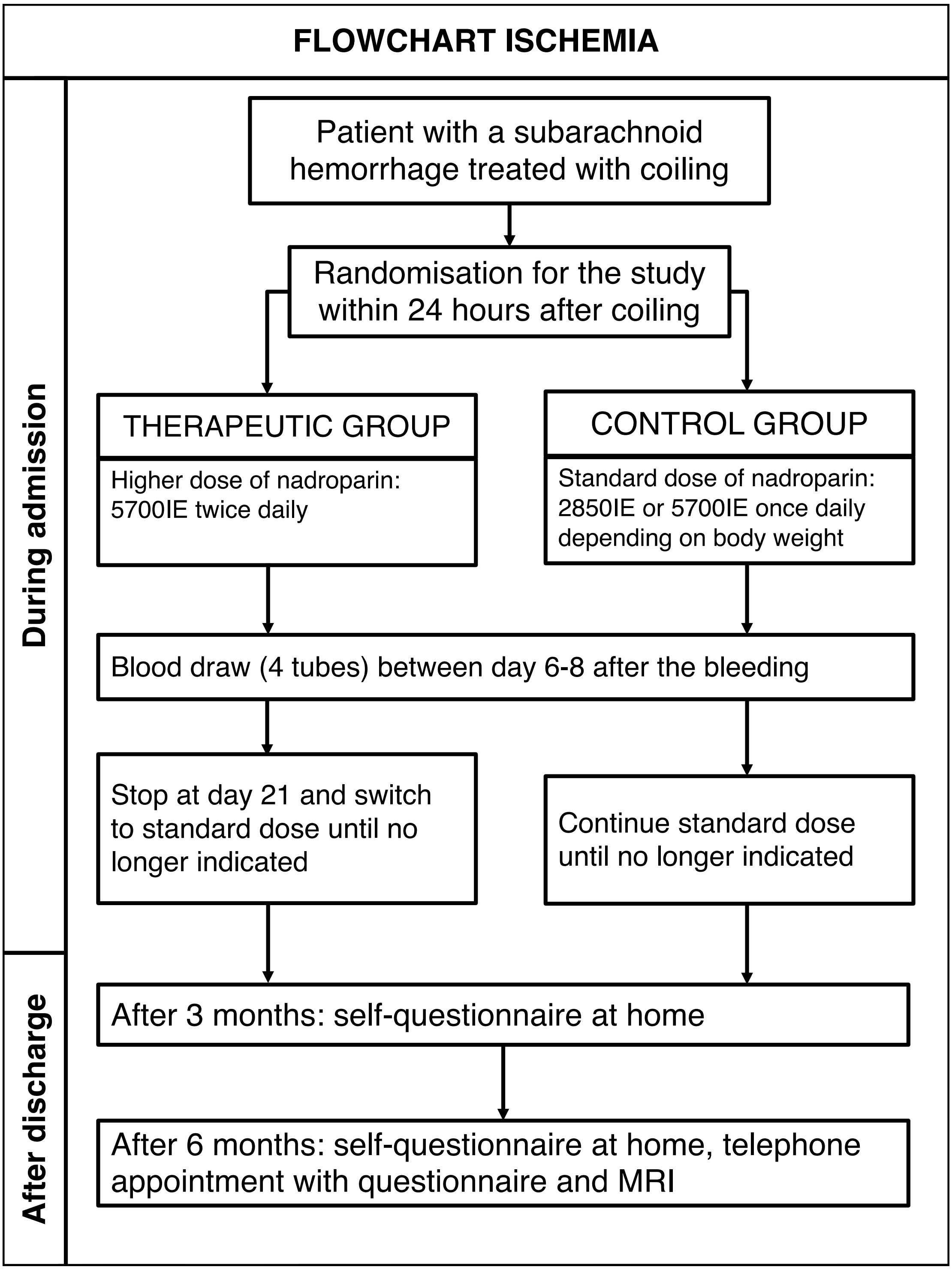

Appendix D – Side effects, adverse effects and disadvantages of nadroparin

As with any drug, nadroparin might lead to side effects in some participants.

Circumstances in which caution is advised

Allergies: these are very rare in people using this drug. Symptoms are:

- Bumps or an itching rash (hives)
- Swelling, sometimes of the face or throat (angioedema), can cause difficulty breathing

Side effects of the drug

Very often (more than 1 out of 10 patients):

- Hemorrhages; these can occur at the site of injection or elsewhere in the body
- Bruising (hematoma) at the site of injection

Often (less than 1 out of 10 patients):

- Redness, swelling at place of injection
- Headache
- Decrease of liver function (increase in certain liver enzymes in the blood)

Rarely (less than 1 out of 1.000 patients):

- Rash, itching and bumps (hives), redness (erythema)
- Calcium deposition in the skin at the site of injection (calcinosis cutis), especially in patients with reduced kidney function
- Blood disorder which results in bruising or increased risk of hemorrhage (thrombocytopenia), or increased number of platelets (thrombocytosis)

Very rarely (less than 1 out of 10.000 patients):

- Death of skin tissue at the site of injection (skin necrosis)
- Hypersensitivity reaction
- Painful and persistent erection of the penis (priapism). If this occurs, you need to inform your physician as soon as possible.
- Migraine
- Increase in certain blood cells (eosinophils) after quitting treatment
- High potassium in the blood (hyperkalemia)

Appendix E: Consent form participant

Concerning the ISCHEMIA study

- I have read the information letter. I could ask additional questions. My questions are sufficiently answered. I had plenty of time to decide whether I would participate or not.
- I know that participation is completely voluntary. I know I can still decide to withdraw or quit the study at any time, without any given reason.
- I give permission to inform my general practitioner about participation in the study.
- I give permission to ask my general practitioner for information regarding my medical health if necessary.
- I give permission to inform my general practitioner or treating physician in case of unexpected findings during the study that could be of importance to my health.
- I give permission to collect and store my data and blood samples. The researchers solely use this data for the goals that are stated in the information letter.
- I know that for the sake of this study some people can see my personal information. Those people are listed in the information letter. I give these people permission to see my personal information for this purpose.
- Would you please fill in the yes/no table below?

I give permission to store my data for the purpose of other studies, as stated in this information letter.	Yes ☐	No ☐
I give permission to use (remaining) blood samples for the purpose of other studies, as stated in this information letter. The blood samples will be stored for 5 years.	Yes ☐	No ☐
I give permission to approach me for further research in the future.	Yes ☐	No ☐
I give permission to request data regarding my official cause of death at the Central Bureau of Statistics in case I die during the course of this study.	Yes ☐	No ☐

- I agree with my participation in the above mentioned study.

My name is (participant): ………………………………..

Signature: ……………………… Date (day/month/year): __ / __ / __

I hereby declare that I have informed this study participant about the above mentioned study.

If new information is becoming available during the study that could affect the participant, I'll inform him/her in a timely manner.

Name investigator (or his/her representative): ……………………………….

Signature: ………………………   Date (day/month/year): __ / __ / __

The participant receives the complete information letter together with a signed version of the consent form.
