# Supplementary material for: Improving outcome in SubaraChnoid HEMorrhage wIth nAdroparin (ISCHEMIA): a prospective randomised controlled trial protocol
Source: BMJ Open. 2025 Aug 28;15(8):e096555. doi: 10.1136/bmjopen-2024-096555 (PMC12410639; doi:10.1136/bmjopen-2024-096555)
Supplement: online supplemental file 2 [file bmjopen-15-8-s002.docx]

Appendix A: Alternative LMWH dose

Table 1 Alternative LMWH dose scheme

| Group | Dalteparine | Enoxaparine |
| --- | --- | --- |
| Low dose | 1dd 2500IE =  1dd 0.1mL of 25000IE/mL syringe | 1dd 20mg =  1dd 0.2mL of 100mg/ml syringe |
| High dose | 2dd 10000IE =  2dd 0.4mL of 25000IE/mL syringe | 2dd 80mg =  2dd 0.8ml of 100mg/ml syringe |
