# Supplementary material for: Improving outcome in SubaraChnoid HEMorrhage wIth nAdroparin (ISCHEMIA): a prospective randomised controlled trial protocol
Source: BMJ Open. 2025 Aug 28;15(8):e096555. doi: 10.1136/bmjopen-2024-096555 (PMC12410639; doi:10.1136/bmjopen-2024-096555)
Supplement: online supplemental file 3 [file bmjopen-15-8-s003.docx]

Appendix B: Flowchart emergency intervention or bleeding


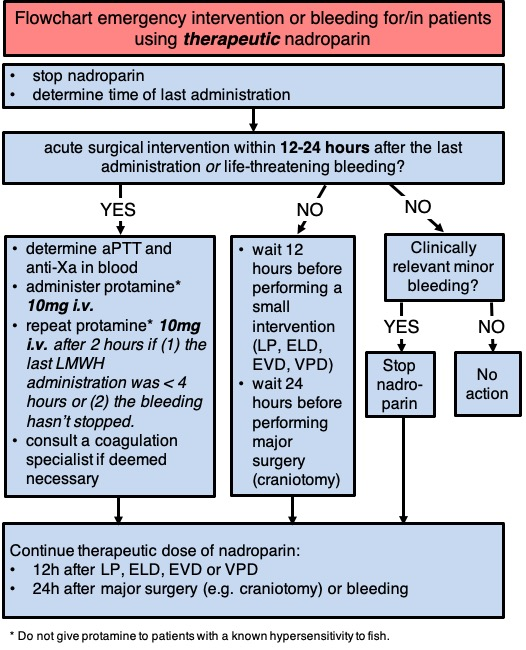


**Figure 1** Flowchart emergency intervention or bleeding (abbreviations: aPTT = activated partial thromboplastin time; LP = lumbar punction; ELD = external lumbar drain; EVD = external ventricular drain; VPD = ventriculoperitoneal drain).
