# Supplementary material for: Improving outcome in SubaraChnoid HEMorrhage wIth nAdroparin (ISCHEMIA): a prospective randomised controlled trial protocol
Source: BMJ Open. 2025 Aug 28;15(8):e096555. doi: 10.1136/bmjopen-2024-096555 (PMC12410639; doi:10.1136/bmjopen-2024-096555)
Supplement: Supplementary file 4 [file bmjopen-2024-096555.reviewer_comments.pdf]

PEER REVIEW HISTORY

BMJ Open publishes all reviews undertaken for accepted manuscripts. Reviewers are asked to complete a checklist review form and are provided with free text boxes to elaborate on their assessment. These free text comments are reproduced below.

ARTICLE DETAILS

Title (Provisional)

Improving Outcome in Subarachnoid Hemorrhage with Nadroparin (ISCHEMIA): A prospective randomized controlled trial protocol.

Authors

Denneman, Nadine; Doorschodt, Tom C; Coert, Bert A; van den Berg, René; Majoie, Charles B. L. M.; Müller, Marcella C.A.; Middeldorp, Saskia; Coppens, Michiel; Kempeneers, Marinus A; Tjerkstra, Maud A; Labib, Homeyra; Bandral, H Verdan; Baarse, Martine; Platek, Ewa E; Vandertop, Peter W.; Verbaan, Dagmar; Post, René

VERSION 1 - REVIEW

**Reviewer**	**1**
**Name**	**Kim, Jennifer**
**Affiliation**	**Yale School of Medicine**
**Date**	**01-Jun-2025**
**COI**	**None**

This study discusses an overall interesting topic in a large cohort of SE patients identified with exposure to PB with a large proportion related to encephalitis who presented with convulsive SE. They noted significant higher use of number of nonanesthetic ASM, duration of anethetics, vasopressors, hospital and NICU LOS in megadose phenobarb patients. The also noted trends in mega dose PB group having poorer SE control, longer days to SE control. Complications were seen in both groups. The topic is likely to be of interest to readers with minor comments to the manuscript:

1) Statistical analysis section is awkwardly worded and can be condensed.

2) Was there any data about whether MDPB patients had longer durations of seizures as an explanation as to why these patients had higher ASM and anesthesia exposure? Even though their baseline demographics show no difference having more explanations as to what is potentially driving this difference would be helpful (e.g., is it something disease severity related, treatment reated or practice variation)

3) Discussion section, while interesting, reports on a lot of tangential literature which could be streamlined/removed.

4) Practice variation, especially across different countries, is highly likely and should be discussed

**Reviewer**	**2**
**Name**	**Busl, Katharina**
**Affiliation**	**University of Florida**
**Date**	**16-Jun-2025**
**COI**	**None**

The authors conduct a single center, non-blinded, randomized clinical trial assessing preliminary efficacy and safety of high- versus low-dose nadroparin after aSAH.

Scientific premise of heparin after aSAH, largely due to anti-inflammatory effects, has been explored in various observational studies, and in a prospective trial, ASTROH, that was halted in 2022 with no results published thus far.

In the outlined trial, there are a number of items that could benefit from clarification.

Introduction: why nadroparin, as opposed to other LMWH or UFH? Please elaborate your scientific basis for this specific choice over others.

Preliminary experience is based on the authors single center observation and retrospective analysis of high-dose nadroparin in a subset of their coiled population. The patient population was heavily tilted to better WNFS grade for high dose nadroparin, and the outcome of mortality biased significantly by WLST, while occurrence of DCI was no different – please elaborate how this served as your preliminary experience paving the way for this phase II trial.

Methods:

Eligibility:

- What does high dose nadroparin correspond to? Is this systemic ac?

- Why no quantify level of anticoagulation by specified Xa measurements?

- Why inclusion of LP diagnosed - --- exceedingly low DCI rate in mF 0 population

- Why is severe HTN a contraindication for LMWH? Can be treated? What is local protocol?

- Is SAH that is aneurysmal but of traumatic origin (with traumatic aneurysm), infectious endocarditis, AVM with aneurysm included? Clarify?

- Do you exclude liver failure with coagulopathy?

Intervention timeline:

- You list as start time of window aneurysm securement via coiling– shouldn't the reference time be the ictus rather than coil date when aiming to homogenize time to DCI window?

- If DCI was no different in your preliminary data – why chose 21 days? With the outlined criteria, it is very unlikely to have many subjects that go until 21 days

Randomization

- How do you plan to balance for WNFS/mF among the enrolled?

Outcomes

- How will you address shift of GOC in the population with the highest expected occurrence of DCI and mortality?

- Why did you not chose objective intermediary outcomes of anticoagulation that would support the primary safety outcome?

- Secondary outcomes: there are a lot, very different ones, and this seems more exploratory and very dependent on local treatment protocols (e.g., how is EVD/LD weaned, who and when are decisions made for shunts)

Blinding- the rationale to not use blinding is weak – could have done placebo and unblind in event of safety occurrence – please explain

Overall assessment:

Preliminary data seem to support trialing heparin for the outlined reason. I am not sure that with this design, a meaningful path to a phase III can be established. I'd further wonder if the authors reached out to the investigators of ASTROH to get insights into why this trial is halted/not completed.

VERSION 1 - AUTHOR RESPONSE

Reviewer #1:
Dr. Jennifer Kim, Yale School of Medicine
Comments to the Author:
This study discusses an overall interesting topic in a large cohort of SE patients identified with exposure to PB with a large proportion related to encephalitis who presented with convulsive SE. They noted significant higher use of number of nonanesthetic ASM, duration of anethetics, vasopressors, hospital and NICU LOS in megadose phenobarb patients. The also noted trends in mega dose PB group having poorer SE control, longer days to SE control. Complications were seen in both groups. The topic is likely to be of interest to readers with minor comments to the manuscript:
1) Statistical analysis section is awkwardly worded and can be condensed.
2) Was there any data about whether MDPB patients had longer durations of seizures as an

explanation as to why these patients had higher ASM and anesthesia exposure? Even though their baseline demographics show no difference having more explanations as to what is potentially driving this difference would be helpful (e.g., is it something disease severity related, treatment reated or practice variation)
3) Discussion section, while interesting, reports on a lot of tangential literature which could be streamlined/removed.
4) Practice variation, especially across different countries, is highly likely and should be discussed

We noted that the comments attributed to Reviewer 1 do not appear to pertain to our submitted manuscript, entitled 'Improving Outcome in Subarachnoid Hemorrhage with Nadroparin (ISCHEMIA): A prospective randomized controlled trial protocol'. The content of Reviewer 1's remarks seems to refer to a different study.

Therefore, in this revision, we have addressed only the comments raised by Reviewer 2, which are relevant to our protocol.

Reviewer #2:
Prof. Katharina Busl, University of Florida
Comments to the Author:
The authors conduct a single center, non-blinded, randomized clinical trial assessing preliminary efficacy and safety of high- versus low-dose nadroparin after aSAH.
Scientific premise of heparin after aSAH, largely due to anti-inflammatory effects, has been explored in various observational studies, and in a prospective trial, ASTROH, that was halted in 2022 with no results published thus far.
In the outlined trial, there are a number of items that could benefit from clarification.
Introduction: why nadroparin, as opposed to other LMWH or UFH? Please elaborate your scientific basis for this specific choice over others.
Preliminary experience is based on the authors single center observation and retrospective analysis of high-dose nadroparin in a subset of their coiled population. The patient population was heavily tilted to better WNFS grade for high dose nadroparin, and the outcome of mortality biased significantly by WLST, while occurrence of DCI was no different – please elaborate how this served as your preliminary experience paving the way for this phase II trial.

We would like to thank the reviewer for their thoughtful and constructive comments on our manuscript. We sincerely appreciate your insightful remarks regarding our study.

Nadroparin was selected primarily for pragmatic reasons. In the Netherlands—and specifically within Amsterdam UMC—nadroparin is frequently used, and its lower dosing regimen constitutes the standard of care in our center. While dalteparin and enoxaparin are equally valid alternatives, and are indeed acknowledged as such in our protocol, fondaparinux was deliberately excluded due to its poor reversibility with protamine.

UFH was not chosen for both practical and theoretical considerations. At our institution, therapeutic titration of UFH can only be performed in the ICU. This would have necessitated prolonged ICU admission (up to three weeks) for all study participants, which was deemed unfeasible. Moreover, achieving stable therapeutic

levels of UFH is notoriously challenging. There is a significant risk of under- or overdosing, as aPTT is not always a reliable indicator of heparin activity in critically ill patients. We were particularly concerned about the risk of overdosing in this vulnerable population. This concern is supported by existing literature (e.g., *van Roessel et al., 2014*, and *Melloni et al., 2008*).

Additionally, one of the central aims of this study is to explore the non-anticoagulant properties of heparins, such as their potential effects on neuroinflammation, demyelination, and apoptosis. These effects are expected to be present in both LMWHs and UFH, as supported by previous findings (e.g., *Hochart et al., 2006*). We have now clarified these considerations in the revised protocol, specifically on page 6 (see track changes).

We acknowledge that in the retrospective cohort, the patient population receiving high dose nadroparin was tilted to better WFNS. However, the observed mortality benefit in the higher-dose nadroparin group remained statistically significant even after adjusting for neurological status at admission. With regard to the comment on potential bias in the mortality outcome due to WLST, we do not entirely agree. The incidence of WLST and brain death was comparable between both study groups.

While we acknowledge that DCI is an important clinical endpoint, it is not the primary outcome of our study. Our main focus is on 30-day mortality. We also agree that high-quality prospective data in this patient population is limited, and that our hypothesis is in part supported by retrospective studies. Given the lack of prospective evidence supporting therapeutic-dose nadroparin in this setting, we considered an RCT to be the appropriate next step. In light of the limited existing data, a phase 2 trial is a reasonable starting point to assess feasibility, safety, and potential efficacy, and to determine whether a full phase 3 trial is warranted. A substantial body of literature supports the potential neuroprotective effects of heparins, and these studies are cited in our protocol. Taken together, we believe this provides sufficient rationale to justify the current phase 2 study, which may provide valuable insights to guide future clinical research in this field.

Methods:
Eligibility:
- What does high dose nadroparin correspond to? Is this systemic ac?

For this study, we chose to use a fixed dose, rather than dosing based on body weight. The dose of 5700 IU twice daily is a therapeutic dose for patients under 70 kg. This protocol was developed in collaboration with a vascular medicine specialist. Most importantly, it reflects that our focus was not solely on the anticoagulant effects of heparin. On the other hand, a fixed dose was chosen to simplify access to the study medication, which becomes more complicated when working with multiple dosing regimens. Furthermore, up until 2021, our hospital-wide protocol was to administer a single weight-independent dose for prophylactic treatment. All patients received 2850 IU once daily, regardless of body weight. Since early 2022, new insights have led to weight-based prophylactic dosing of nadroparin: 2850 IU once daily for patients under 100 kg, and 5700 IU once daily for patients over 100 kg. The rationale for this choice has now been added to the manuscript, on page 10.

- Why no quantify level of anticoagulation by specified Xa measurements?

Because we are focusing on multiple properties of heparin and not solely on its anticoagulant effect, we did not report a cut-off value in the previous version of the manuscript. However, peak anti-Xa levels were measured in the therapeutic group to explore as a potential determinant of increased bleeding risk. Of note, target anti-Xa levels for nadroparin are unknown. Based on observed anti-Xa levels in subjects with a therapeutic weight-based dose, reference ranges are regularly reported (usually around 0,6-1,2 IU/ml) but it remains uncertain whether benefits and harms of nadroparin are optimally maintained in this range (Boneu et al., 1998).

- Why inclusion of LP diagnosed - --- exceedingly low DCI rate in mF 0 population

We aimed to include the most comprehensive aneurysmal SAH population possible and deemed safe. This is in accordance with the diagnostic criteria stated in the Dutch guideline and the protocol used, for example, in the ULTRA trial. However, since the ictus in these patients is often > 72 hours, n=0 in our cohort so far.

- Why is severe HTN a contraindication for LMWH? Can be treated? What is local protocol?

Severe, persistent hypertension was defined as an exclusion criterion for safety reasons, due to the significantly increased risk of intracranial hemorrhage associated with high-dose nadroparin. In such cases, patients are typically transferred to the ICU for intravenous labetalol treatment. However, no patients have been excluded on the basis of this criterion to date.

- Is SAH that is aneurysmal but of traumatic origin (with traumatic aneurysm), infectious endocarditis, AVM with aneurysm included? Clarify?

Patients with traumatic aneurysms were not included in the study, nor have any such patients presented since the study began. Infective endocarditis is already listed as an exclusion criterion. Patients with AVMs were also not included, primarily because AVMs are generally not treated with coiling—except in cases where an intranidal aneurysm is the source of bleeding. However, no such cases have occurred during the study period. We have clarified the in- and exclusion criteria on pages 7-9.

- Do you exclude liver failure with coagulopathy?

We did not specifically exclude liver failure with coagulopathy. However, we do exclude patients with a history of hemorrhagic diathesis due to coagulation disorders (with the exception of disseminated intravascular coagulation), which encompasses various coagulopathies. This exclusion list was developed in collaboration with a vascular medicine specialist and is consistent with the information provided by the Dutch 'pharmacotherapeutic compass' (Farmacotherapeutisch Kompas Nadroparine).

Intervention timeline:
- You list as start time of window aneurysm securement via coiling– shouldn't the reference time be the ictus rather than coil date when aiming to homogenize time to DCI window?

For safety reasons, we administer high-dose nadroparin only to patients with a secured aneurysm. The current inclusion criteria take both the time of ictus and the coiling date into account. Only patients who undergo coiling within 72 hours of the initial SAH are eligible, with most starting nadroparin within 24 hours after coiling. In practice, the majority of included patients present on the day of ictus or the following day and are coiled within 24 hours. Since DCI typically occurs between days 4 and 10, we considered this a suitable treatment window.

- If DCI was no different in your preliminary data – why chose 21 days? With the outlined criteria, it is very unlikely to have many subjects that go until 21 days
That is indeed a valid concern and represents a challenge we frequently encounter during the study, as many patients are discharged between days 14 and 21. The rationale for this study originated from the observed difference in mortality in our retrospective cohort, which lacked a clear explanation. In this context, DCI is an important factor to investigate. Although DCI rates did not significantly differ between groups in the retrospective data, this may be attributed to other factors such as suboptimal documentation. At our center, patients with aSAH remain hospitalized for a minimum of 14 days. While most cases of DCI occur between days 4 and 10 following the ictus, we do occasionally observe instances of late-onset DCI, around day 18, although these are uncommon. In the study by Bruder et al., heparin was administered for only 7 days. To minimize the risk of undertreatment or insufficient duration of treatment, we pragmatically selected a 21-day treatment period (or until hospital discharge, if earlier) to ensure adequate coverage of the DCI window.

Randomization
- How do you plan to balance for WNFS/mF among the enrolled?

In our study design we stratified randomization based on the WFNS. WFNS reflects disease severity and is associated with our primary endpoint, mortality. Stratifying by WFNS helps to ensure a balanced randomization within the different strata based on disease severity. We also collect the modified Fisher scale, but we did not use it for stratification. Most patients with aSAH present with a modified Fisher grade of 4, limiting its discriminative value in the context of stratification.

Outcomes
- How will you address shift of GOC in the population with the highest expected occurrence of DCI and mortality?

We recognize that in patients with aSAH, particularly those with high-grade hemorrhage (e.g., WFNS IV-V), GOC may shift toward comfort-only measures. Such shifts can independently influence mortality outcomes, irrespective of the intervention under investigation. At our center, however, we follow an expeditious treatment approach. Nearly all patients, including those with high WFNS scores, undergo prompt aneurysm occlusion and placement of an external ventricular drain when indicated. WOLS decisions are often not made in the initial phase but only after a lack of improvement despite maximal treatment efforts. This approach similarly applies to DCI, where we implement multiple preventive (e.g., maintenance of normovolemia, administration of nimodipine) and therapeutic (e.g., induced hypertension in the ICU)

strategies. Consequently, early WOLS is uncommon in our cohort. To mitigate this potential source of bias, we stratify randomization by WFNS grade. Additionally, because all decisions regarding care limitations are meticulously documented, sensitivity analyses can be conducted to exclude patients in whom active treatment was withdrawn or significantly limited within the first 72 hours post-randomization—those who may not have had a meaningful chance to benefit from the intervention. Multivariable analyses will also be performed, allowing early WOLS to be considered as a potential confounding variable. We have also clarified this on pages 9 and 14.

- Why did you not chose objective intermediary outcomes of anticoagulation that would support the primary safety outcome?

We did collect these data, but we fully acknowledge that they were not clearly described in the manuscript. Anti-Xa levels are measured on day 7 following the ictus and are readily available. Additionally, we are collecting a comprehensive panel of coagulation and inflammatory markers, which will be analyzed upon completion of patient inclusion. These samples are stored in our biobank and will be processed in a single batch. Specifically, the markers include von Willebrand factor, syndecan, soluble thrombomodulin (sTM), TNF, IL-6, IL-8, soluble VCAM, angiopoietin, ADAMTS13, D-dimer, and Coagulation Factor III (Tissue Factor), all measured on day 7 post-ictus. We have added this information to page 12 of the revised manuscript.

- Secondary outcomes: there are a lot, very different ones, and this seems more exploratory and very dependent on local treatment protocols (e.g., how is EVD/LD weaned, who and when are decisions made for shunts)

We acknowledge that the secondary outcomes are diverse and that some may be subject to variation based on local clinical practices. Nevertheless, they were included to provide a broad and clinically relevant assessment of potential effects and complications associated with aSAH management. We view these outcomes as exploratory and will interpret them with appropriate caution. Notably, the majority of secondary endpoints are collected using standardized definitions, and key clinical decisions—such as EVD/LD weaning and shunt placement—are guided by established protocols and aligned, where feasible, with current consensus recommendations (e.g., Hoh et al., 2023). These limitations will be transparently addressed in our analysis and discussion of the results.

Blinding- the rationale to not use blinding is weak – could have done placebo and unblind in event of safety occurrence – please explain

We thank the reviewer for this important comment. While we recognize that a placebo-controlled, blinded design is the gold standard to minimize bias, we opted for an open-label approach due to both practical and ethical considerations. Nadroparin carries a risk of bleeding, and early recognition of hemorrhagic complications is essential. Unblinding in response to clinical deterioration may delay critical decisions or lead to uncertainty in management. Moreover, nadroparin has measurable pharmacodynamic effects (e.g., anti-Xa levels), which would likely unblind treating clinicians regardless. Implementing placebo with sham anti-Xa monitoring would be ethically and logistically challenging in this vulnerable population. Administering a placebo while simulating anti-Xa monitoring would require drawing blood or performing laboratory assessments

purely for the purpose of maintaining blinding, without any therapeutic benefit. In a critically ill population, this exposes patients to unnecessary interventions, which raises ethical concerns. To reduce bias, we included a blinded trial nurse and selected a primary outcome (mortality) that is objective and not susceptible to observer bias. Nonetheless, we fully acknowledge the lack of full blinding as a limitation, as was explicitly noted in the manuscript.

Overall assessment:
Preliminary data seem to support trialing heparin for the outlined reason. I am not sure that with this design, a meaningful path to a phase III can be established. I'd further wonder if the authors reached out to the investigators of ASTROH to get insights into why this trial is halted/not completed.
We contacted the ASTROH investigators on multiple occasions but, unfortunately, did not receive a response regarding the reason for the study's discontinuation. We would like to thank the reviewer for their thoughtful feedback and hope that our responses have adequately addressed the concerns raised.

VERSION 2 - REVIEW

**Reviewer**	**2**
**Name**	**Busl, Katharina**
**Affiliation**	**University of Florida**
**Date**	**30-Jul-2025**
**COI**	

Thank you for addressing my comments.

I would suggest to add some of the replies you added to the reply document but not to the manuscript to the actual manuscript, for clarity and to show you thought of this 1) randomization strata, 2) timing of nadropin within 96 hrs, 3) reasons for unblinded 4) choice of intermediate outcome (mortality) and what your go- no-go criteria will be for suggesting a phase 3. what is a significant reduction in mortality? statistic result versus clinically relevant?

VERSION 2 - AUTHOR RESPONSE

Reviewer #2:
Prof. Katharina Busl, University of Florida
Comments to the Author:
Thank you for addressing my comments.
I would suggest to add some of the replies you added to the reply document but not to the manuscript to the actual manuscript, for clarity and to show you thought of this 1) randomization strata, 2) timing of nadropin within 96 hrs, 3) reasons for unblinded 4) choice

of intermediate outcome (mortality) and what your go- no-go criteria will be for suggesting a phase 3. what is a significant reduction in mortality? statistic result versus clinically relevant?

Additional details on the randomization strata have been included on pages 8–9. Information regarding the timing of nadroparin administration has been added to the *Interventions* section on page 9. The rationale for unblinding and the selection of mortality as the primary endpoint are now thoroughly explained on page 17. Clinically meaningful mortality thresholds for advancing to a larger phase III trial are elaborated on page 16.

VERSION 3 - REVIEW

**Reviewer**	**2**
**Name**	**Busl, Katharina**
**Affiliation**	**University of Florida**
**Date**	**15-Aug-2025**
**COI**	

---

all comments addressed

of note - this is a steep improvement in mortality as a go-no-go, and possible bias through WLST (or avoidance of WLST) will have to be addressed
